# The Neuroprotective Role of Acorus calamus in Developmental and Histopathological Changes in Autism-Induced Wistar Rats

**DOI:** 10.7759/cureus.29717

**Published:** 2022-09-28

**Authors:** Kavitha Ukkirapandian, Kayalvizhi E, Karthika Priyadharshini Udaykumar, Suganya Kandhi, Muthulakshmi R

**Affiliations:** 1 Physiology, Meenakshi Medical College Hospital and Research Institute, Meenakshi Academy of Higher Education and Research (MAHER) University, Chennai, IND; 2 Physiology, Sri Venkateshwaraa Medical College Hospital and Research Centre, Pondicherry, IND; 3 Physiology, Sri Ramachandra Institute of Higher Education and Research, Chennai, IND

**Keywords:** neural reflexes, histopathology, valproate, acorus calamus, rat model of autism

## Abstract

Introduction

Autism spectrum disorder (ASD) is a neurodevelopmental disorder, and a tremendous increase in the incidence of autism poses challenges in identifying the different treatment modalities. Since the defined etiology, pathophysiology, and treatment of autism are unavailable, translational research is being done by creating animal models of autism. This study aimed to assess the effects of *Acorus calamus *on developmental and histopathological changes in autism-induced Wistar rats.

Materials and methods

A rat model of autism was created by administering sodium valproate on the 12th day of pregnancy, and rat pups of this group were considered autism-induced. Rat pups of pregnant rats who had received normal saline on the 12th day of pregnancy were considered group I (negative control group). Neural reflexes were assessed in early postnatal days (PND) to confirm the development of autism. Autism-induced rat pups were divided into the following two groups: group II, autism (positive control group), and group III, autism + *A. calamus* (drug-treated group). On the 21st postnatal day (PND), group III was given an ethanolic extract of *A. calamus* (200 mg/kg), and group I and group II were given normal saline orally for 15 days. After 15 days of drug exposure, at 36thPND, the rats were sacrificed, and brain tissue was collected for histopathological analysis.

Results

When compared to the negative control group, autism-induced rat pups showed delayed appearance of neurological reflexes. Neurodegenerative changes were well appreciated in group II (autism-induced rats) than in group III (autism + *A. calamus*). In the histomorphometric analysis, group II showed a significant reduction in the number of neurons in the frontal cortex and Purkinje cells in the cerebellum. However, when compared to group II, group III (autism treated with *A. calamu*s) did not show significant alteration.

Conclusion

Valproate exposure at mid-pregnancy creates autism by disturbing neural structures among rat pups. This was clinically represented as the delayed appearance of neural reflexes. *Acorus calamus* in the early postnatal period protects rat pups’ brain morphology against autism pathology.

## Introduction

Autism spectrum disorder (ASD) includes a group of developmental disabilities, which is usually manifested as impairment in motor development, language development, and behavioral abnormalities. The incidence of ASD globally is about one in 160 children [1]. The incidence rate of autism is higher in developed countries than in other geographical areas. It may be due to a lack of awareness. There is no well-defined treatment for autism, so multiple therapeutic approaches were followed at the earlier stage for a better prognosis.

Animal models of autism were created to explore the hidden fact behind autistic pathology. Although autism is mainly related to gene incompatibility, there are genetic and non-genetic models of autism. Among animal models of autism, prenatal valproate-induced autism has shown face, construct, and predictive validity [2]. Valproate is an anticonvulsant drug, and it is used prophylactically to manage epilepsy during pregnancy. However, cases reported that valproate is a potent teratogen, and large single doses of valproate reach peak levels in the fetal serum, thus resulting in congenital defects [3]. Intrauterine exposure to valproic acid just before neural tube closure (12th day of embryonic period) creates behavioral and biochemical changes among rat pups that mimic the autism pathology. Previous studies reported that the prenatal valproate model of autism, which mainly resembles human autistic features, was the widely accepted animal model of autism [4].

*Acorus calamus*, commonly known as Vacha, is traditionally used in Indian and Chinese ayurvedic medicine, and its rhizome part is used as one of the compounds of pharmaceutical products. Previous scientific studies proved that *A. calamus* has anticonvulsant action and anti-stress and antioxidant activity. In human studies, the rhizome part of *A. calamus*, used in treating Alzheimer’s disease and schizophrenia, has demonstrated improvement in symptoms. Animal studies on rats reported that the rhizome part of *A. calamus* has neuroprotective effects [5,6].

Hence, this study was carried out to explore the neuroprotective effects of *A. calamus* on developmental and histopathological changes among valproate-induced autism rats.

## Materials and methods

This experimental study was undertaken in the animal house and central research laboratory of Meenakshi Medical College after obtaining clearance from the Institutional Animal Ethical Committee (IAEC). The protocol clearance number was IEAC 001/2019. The experiments were conducted by following the Committee for the Purpose of Control and Supervision of Experiments on Animals (CPCSEA) guidelines. Pregnant rats were purchased from MASS BIOTECH (Chettipunyam, India) (registration number 2084/PO/Bt/S/19/CPCSEA). Following CPCSEA guidelines, animals were housed in an institutional animal house.

Autism induction and grouping

A rat model of autism was created by administering sodium valproate (600 mg/kg/IP) on the 12th day of pregnancy [7]. Rat pups of pregnant rats who received normal saline on the 12th day of gestation were considered group I (n=6) (negative control group), and rat pups of pregnant rats exposed to valproate were considered group II (autism-induced group) (n=6). All pregnant rats delivered at the end of 28 days around, and the average litter size in our study was five.

Neural reflexes were assessed in early postnatal days (PND) to assess the development of autism in both groups. Righting and cliff avoidance reflexes were assessed at PND 3 and PND 7. Righting reflex was assessed by placing the rat offspring in a supine position; the time taken to correct its posture was noted as 15 seconds. Cliff avoidance reflex was assessed by placing the rat offspring over the edge, and the protective response, where the rat pup turns away from the edge of the cliff, was noted. Scoring was done manually: 0 for no movement or falling off the edge, 1 for attempts to move away from the cliff but with hanging limbs, and 2 for successful movement away from the cliff [8].

On 21st PND, group I rat pups were given normal saline (negative control + normal saline), whereas on 21st PND, autism-induced rat pups were divided into the following two groups: group II, autism (positive control group + normal saline), and group III, autism + *A. calamus *(drug-treated group). Each group consists of 10 rat pups.

Preparation of ethanol extract and drug administration

The rhizome part of *Acorus calamus* was collected from the cultivation area, and authentication was obtained from a botanist at the Council of Scientific and Industrial Research (CSIR), Siddha Institute, Chennai, India. Course powder was made using a mechanical grinder. Course powder of *A. calamus* was mixed with 90% ethanol, and ethanol extract of *A. calamus* was prepared using the Soxhlet apparatus. The ethanol extract was concentrated using a hot water bath.

Ethanol extract of *A. calamus* was mixed with 0.5% carboxymethylcellulose (CMC); CMC was used here as a vehicle. The clinically effective dose of *A. calamus* for human beings is 1,000-1,500 mg/60 kg. It was around 25 mg/kg.

*Acorus calamus* dosage per kg of body weight for rat pups was calculated using the following formula: animal dose (mg/kg) = human dose (mg/kg) × conversion factor. The conversion factor for human versus rat is 6.2; therefore, the calculated animal dose of *A. calamus* is nearly 200 mg/kg [9].

Ethanol extract of *A. calamus* (200 mg/kg) was given orally for 15 days to see the acute effects of *A. calamus*. Drug administration was started on the 21st postnatal day, which is the weaning age of rats [10].

Animal dissection

After the drug course, all the animals were given pentobarbital 40 mg/kg/IP to induce anesthesia [11], and the animals were dissected at PND 36. Brain tissue was collected in 10% formalin for histopathological analysis. Sagittal sectioning was done at the following levels: (1) optic chiasm, (2) median eminence, and (3) midway cerebellum.

Hematoxylin and eosin (H&E) staining was done. Histomorphometry analysis was done to measure the mean number of viable neurons (prefrontal cortex) and the number and diameter of Purkinje cells (cerebellum) in H&E-stained sections using the image analyzer MagVision (Magnus Opto Systems India Pvt. Ltd., New Delhi, India) digital software installed on LX-300 (Labomed Laboratory Microscope, Labo America, Inc., CA, USA). The MagVision image analyzer software was first calibrated using a stage microscope with standard measurement (1 unit = 0.01 mm).

Statistical analysis

Data were analyzed using GraphPad Prism (8.4.2) (GraphPad Software, California, USA). Student’s unpaired t-test was applied to compare the control and autism-induced groups. One-way analysis of variance (ANOVA) was used to find a statistical difference between the three groups (control, autism, and autism + *A. calamus*). P-value < 0.05 was considered significant.

## Results

Student’s t-test revealed a P-value of 0.001 when comparing the latency of righting reflex on the third postnatal day between the autism-induced group (mean: 13.86 seconds) and the control rat pups (mean: 6 seconds). In the autism-induced group, the mean score of the cliff avoidance reaction was 1.16 on the seventh postnatal day, while it was 2.5 in the control group (Table 1).

**Table 1:** Neural reflexes Latency of righting reflex and score of cliff avoidance reflex were presented here as mean ± SD (x ± y). SD: standard deviation

Newborn rat reflexes	Control (mean ± SD)	Autism-induced group (mean ± SD)	P-value
Righting reflex (seconds)	6.00 ± 0.30	13.83 ± 0.36	<0.000
Cliff avoidance reflex (score)	2.5 ± 0.15	1.16 ± 0.11	<0.000

**Figure 1:**
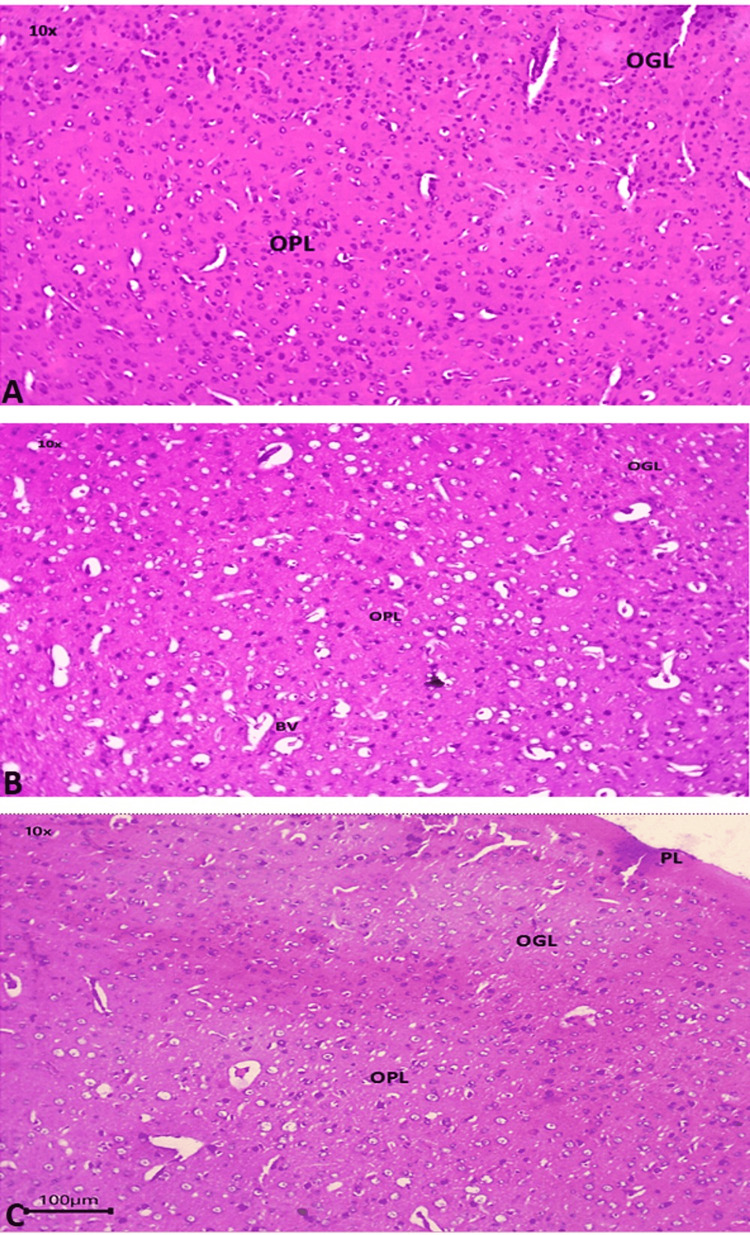
Histopathological changes: prefrontal cortex (10×) 1A: group I (control); 1B: group II (autism induced); 1C: group III (autism induced + *A. calamus*) OGL: outer granular layer; OPL: outer pyramidal layer; BV: blood vessels; PL: pyramidal layer

Figures 1A, 1B, 1C depict the outer granular layer and the outer pyramidal layer of the prefrontal cortex at low power (10×). Granular and pyramidal layers in group I (control) were healthy, and the number of brain cells was ideal (Figure 1A). However, more vacuoles were seen in group II (autism induced), indicating neural degeneration (Figure 1B). Group III (autism induced + *A. calamus*) had fewer vacuoles when compared to group II (Figure 1C).

**Figure 2:**
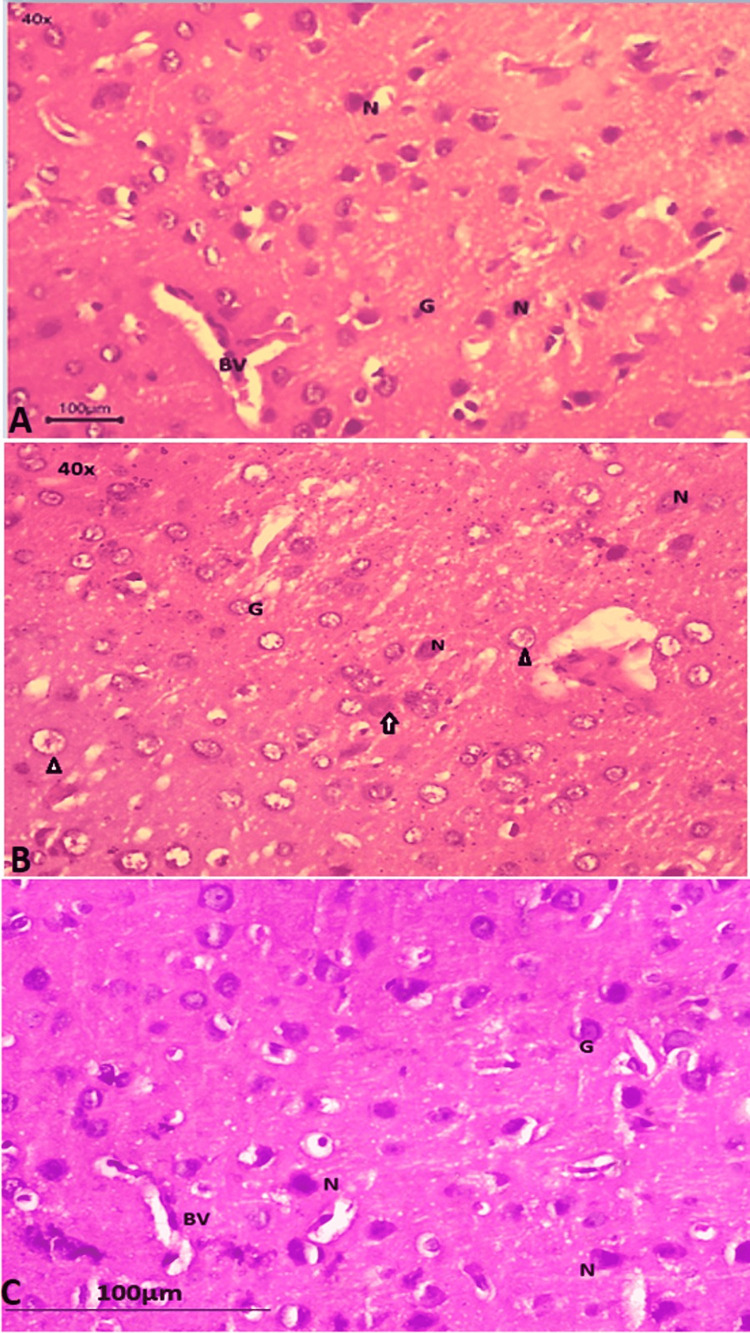
Prefrontal cortex (40×) 2A: group I (control); 2B: group II (autism induced); 2C: group III (autism induced + *A. calamus*) N: neuronal cell; G: glial cell; BV: blood vessels; black arrowhead: granulovacuolar degeneration of the neuron cells; black arrow: neuron cell with chromatolysis

Pyramidal neuron cells and glial cells were seen in the prefrontal cortex at high magnification (40×) in Figures 2A, 2B, 2C. In group I (control), there was no sign of chromatolysis or degeneration, and the morphology of the neurons was normal (Figure 2A). In group II (autism induced), the morphology of the neurons was altered, which indicates granulovacuolar degeneration of neural cells, and chromatolysis was evident (Figure 2B). The greatest number of neurons in group III (autism induced + *A. calamus*) were spared structural deformities (Figure 2C).

**Figure 3:**
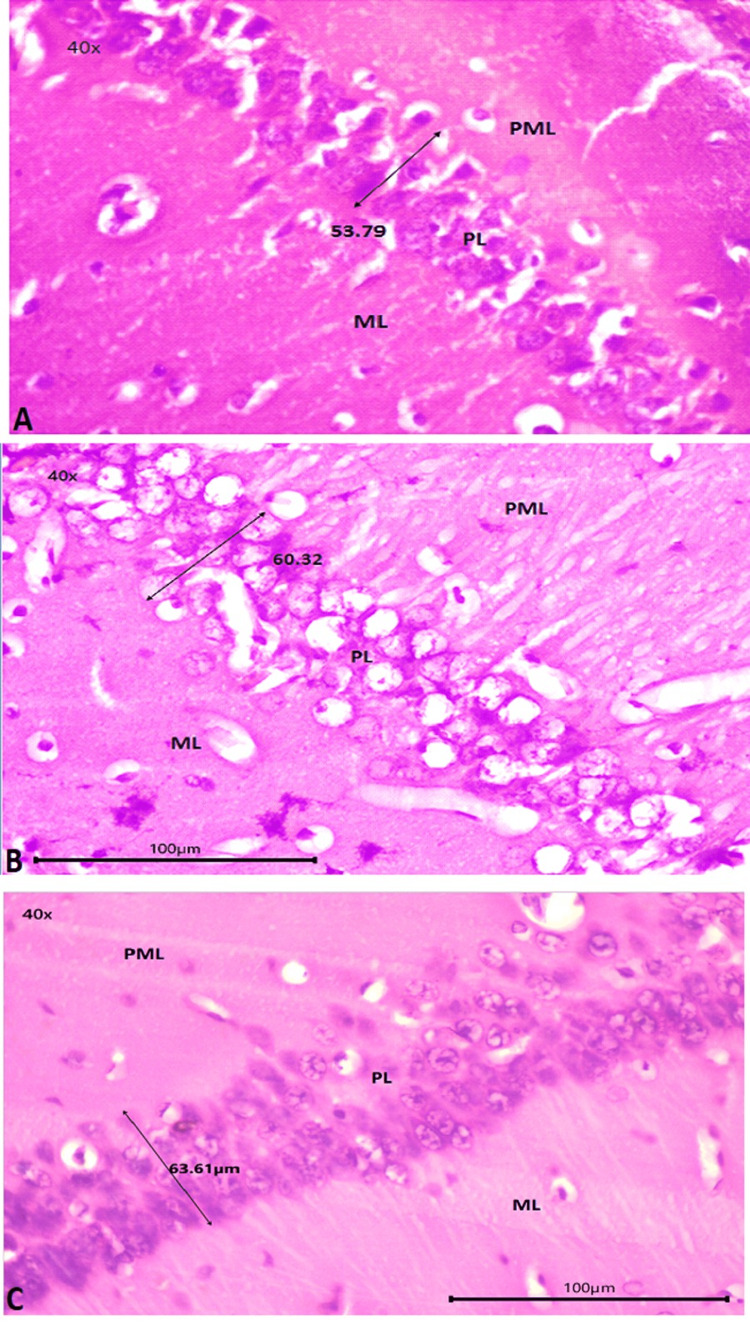
Hippocampus (40×) 3A: group I (control); 3B: group II (autism induced); 3C: group III (autism induced + *A. calamus*) PML: polymorphic layer; PL: pyramidal layer; ML: molecular layer; measurement arrow: skewing of the pyramidal layer

Figures 3A, 3B, 3C show the polymorphic layer, pyramidal layer, and molecular layer in the CA1 area of the hippocampus at high magnification (40×). In group I (control), the thickness of the pyramidal layer was unaffected, and the morphology of all three layers was normal (Figure 3A). Pyramidal cells in the pyramidal layer were affected in group II (autism induced), as evidenced by the presence of vacuoles, and the pyramidal layer was distorted (Figure 3B). Compared to group I, group III (autism induced + *A. calamus*) had damaged pyramidal cells (Figure 3C) but is not affected as group II.

**Figure 4:**
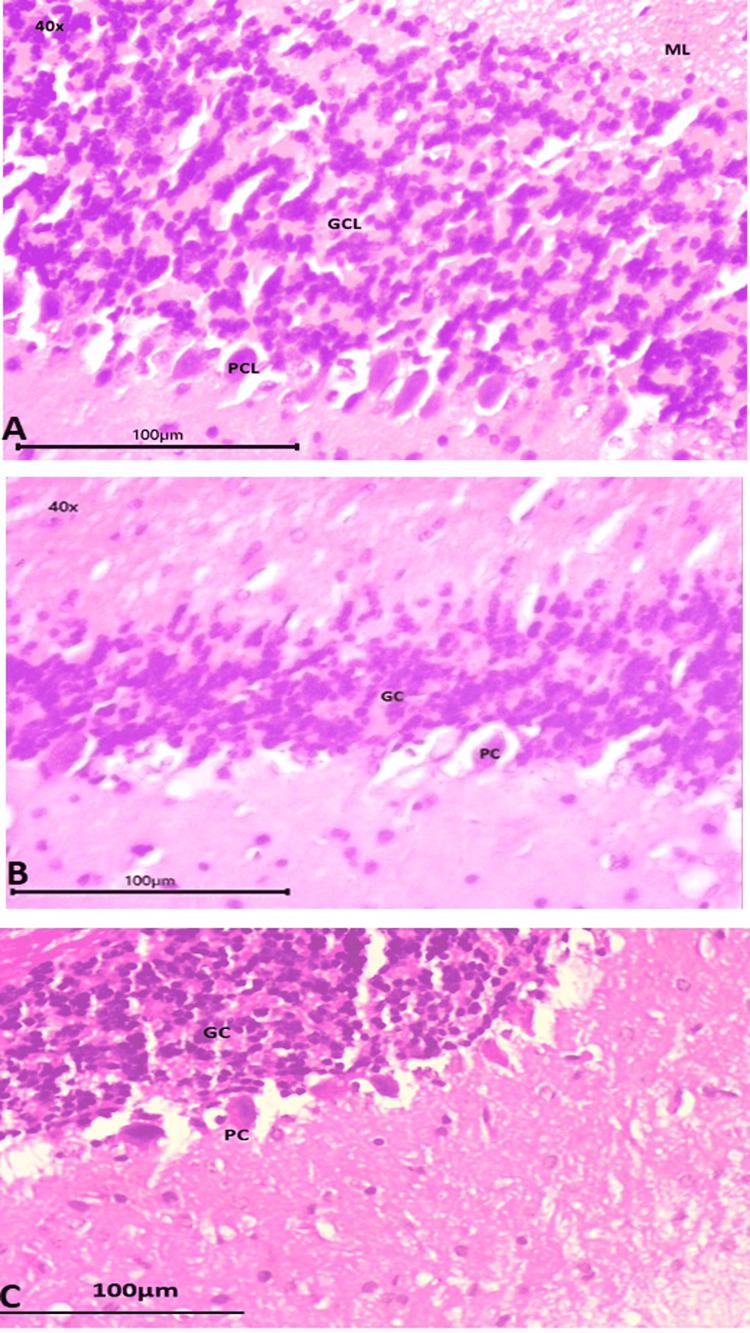
Cerebellum (40×) 4A: group I (control); 4B: group II (autism induced); 4C: group III (autism induced + *A. calamus*) GC: granular cell; PC: Purkinje cell; ML: molecular layer; GCL: granular cell layer; PCL: Purkinje cell layer

In Figures 4A, 4B, 4C, the cerebellum showed a molecular layer, granular cell layer, and Purkinje cell layer at high power (40×). Group I had the thickest granular layer compared to the other groups, and it also had the most Purkinje cells (Figure 4A). Group II had hypocellularity in both Purkinje and granular cell layers. Purkinje cells were also anucleated in group II (Figure 4B), whereas they were entirely fully nucleated in group III, despite some hypocellularity (Figure 4C).

The mean numbers of neurons in the prefrontal cortex, the mean number of Purkinje cells, and the diameter of Purkinje cells were compared between all three groups (Table 2). The numbers of neurons and Purkinje cells were significantly reduced (one-way ANOVA) in group II than in the other two groups. The diameter of Purkinje cells was also reduced in group II when compared to group I and group III.

**Table 2:** Histomorphometric analysis Data were presented here as mean ± SD (x ± y). SD: standard deviation

	Group I (mean ± SD)	Group II (mean ± SD)	Group III (mean ± SD)	P-value
Mean number of viable neurons in the prefrontal cortex (numbers)	28.25 ± 1.31	14.25 ± 1.11	18.25 ± 1.31	<0.0001
Mean number of viable Purkinje cells (numbers)	12.25 ± 0.85	4.75 ± 0.48	7.75 ± 0.48	<0.0001
Diameter of Purkinje cells (µm)	7.69 ± 0.25	7.42 ± 0.29	7.66 ± 0.25	0.89

## Discussion

In this study, neural reflexes righting and cliff avoidance reflexes were appreciated, which were delayed in the autism-induced group when compared to the control group. These findings demonstrated the developmental delay in autism. Valproate, an anticonvulsant drug, inhibits gamma-aminobutyric acid (GABA) transaminase by increasing the concentration of GABA. Additionally, it also acts on GABA receptors to potentiate the postsynaptic effect [12]. Valproate also reduces the neural expression of glutamate receptors [12,13]. The imbalance between excitatory and inhibitory neurotransmitters during the developmental stage of the rat fetus results in autism-like symptoms, which delay neural reflexes.

Other than the therapeutic effect, the carboxylic acid moiety of valproate has been linked with neural tube defects. At physiological pH, carboxylate (COO-), as an anion, interacts with organic anion transporters present in the placenta. Due to this interaction, the level of valproate increases in the fetus, which results in embryotoxicity. Besides, valproate has a high affinity for protein, so the clearance rate is minimal [14].

Histopathological analysis revealed vacuole appearance in neurons and chromatolysis in the prefrontal cortex of autism-induced rats. The prefrontal cortex is mainly responsible for higher-order functioning and structural alteration in neurons, which results in disordered social behavior (a peculiar feature of autism). The pyramidal layer in the hippocampus CA1 region was disturbed, and pyramidal cells were disintegrated in autism-induced rats. The hippocampus plays a major role in controlling emotional components by maintaining a neural circuit with the frontal cortex. So, alteration in pyramidal cells and their circuit would be the reason for emotional instability in autistic children. Hypocellularity in the granular layer and Purkinje cell layer were appreciated in autism-induced rats. The cerebellum is mainly involved in postural reflexes and equilibrium. This cerebellum pathology involvement is also the reason for the delayed appearance of reflexes demonstrated in the early age of autism rats [15]. The delayed appearance of reflexes and cerebellum involvement were similar to the clinical features seen in autistic children.

The above-reported pathological changes in autism-induced rats are mainly due to the genomic action of valproate on histone deacetylase (HDAC). HDAC promotes histone to wrap DNA tightly and maintain its structure. When this enzyme gets inhibited by valproate, it results in altered gene transcription and the alteration of neuronal synopsis [15]. Histomorphometric results in this study also support autism pathology similar to autism in children.

*Acorus calamus* is a well-known medicinal plant. Numerous scientific studies have been conducted both in animals and human beings to demonstrate its various effects, such as anti-inflammatory, antidiabetic, cardioprotective, and neuroprotective effects [5]. Esfandiari et al. (2018) mentioned that extracts of *A. calamus* prevent memory loss, anxiety, and oxidative stress among rats exposed to lipopolysaccharide-induced neuroinflammation [16]. Previous studies reported that *A. calamus* extract improved behavioral patterns in neuropathic rat models [17], and it also showed neuroprotective action against ischemia-induced (cerebral artery occlusion) rats [18].

The secondary metabolites of the rhizome part of *A. calamus* are mainly α- and β-asarone. Both α- and β-asarone exhibit multiple pharmacological properties, including antioxidant, anti-inflammatory, anti-apoptotic, and neuroprotective effects by crossing the blood-brain barrier [19]. β-Asarone protects cognitive function by inhibiting ACh esterase and suppressing tumor necrosis factor-α (TNF-α) and interleukin-1β (IL-1β) secretion among Alzheimer’s disease-induced rats [6,20]. α-Asarone showed anticonvulsant activity in seizure-induced animal models and by its GABA antagonist and N-methyl-D-aspartate (NMDA) receptor agonist properties [21]. The pharmacological action of α- and β-asarone would be the reason for significant neuroprotective effects on autism pathology among rat pups treated with *A. calamus*.

This current study demonstrated that *A. calamus* as a whole herbal plant protects the anatomical structures from autism pathology. However, the limitation of this current study was as follows: (1) extensive biochemical analysis could have been done to support the histopathological changes, and (2) different phytochemicals present in* A. calamus* should also have been used separately to identify the mechanism of action further. Since this study is the initial step to knowing the action of *A. calamus* on autism, the abovementioned limitations will be rectified by extending the work further in the future.

## Conclusions

This study showed developmental and histopathological changes in valproate-induced autism and the positive effects of *A. calamus* by producing neuroanatomical changes. *Acorus calamus*, with its antioxidant, anti-inflammatory, GABA agonist, and cholinergic actions, protects neural structures among autism-induced rats. Management of autism is still challenging since complex neuronal structures are involved. *Acorus calamus* as a whole medicinal plant exerts its positive effects by acting on multiple pathways. Hence, *A. calamus* can be given importance to study further to identify the exact mechanism of action in treating autism.
